# PPARγ Inhibits VSMC Proliferation and Migration via Attenuating Oxidative Stress through Upregulating UCP2

**DOI:** 10.1371/journal.pone.0154720

**Published:** 2016-05-04

**Authors:** Yi Zhou, Ming-Jie Zhang, Bing-Hu Li, Lei Chen, Yan Pi, Yan-Wei Yin, Chun-Yan Long, Xu Wang, Meng-Jiao Sun, Xue Chen, Chang-Yue Gao, Jing-Cheng Li, Li-Li Zhang

**Affiliations:** Department of Neurology, Institute of Surgery Research, Daping Hospital, Third Military Medical University, 10 Changjiang Branch Road, Yuzhong District, Chongqing, 400042, PR China; University of Sassari, ITALY

## Abstract

Increasing evidence showed that abnormal proliferation and migration of vascular smooth muscle cells (VSMCs) are common event in the pathophysiology of many vascular diseases, including atherosclerosis and restenosis after angioplasty. Among the underlying mechanisms, oxidative stress is one of the principal contributors to the proliferation and migration of VSMCs. Oxidative stress occurs as a result of persistent production of reactive oxygen species (ROS). Recently, the protective effects of peroxisome proliferator-activated receptor γ (PPARγ) against oxidative stress/ROS in other cell types provide new insights to inhibit the suggests that PPARγ may regulate VSMCs function. However, it remains unclear whether activation of PPARγ can attenuate oxidative stress and further inhibit VSMC proliferation and migration. In this study, we therefore investigated the effect of PPARγ on inhibiting VSMC oxidative stress and the capability of proliferation and migration, and the potential role of mitochondrial uncoupling protein 2 (UCP2) in oxidative stress. It was found that platelet derived growth factor-BB (PDGF-BB) induced VSMC proliferation and migration as well as ROS production; PPARγ inhibited PDGF-BB-induced VSMC proliferation, migration and oxidative stress; PPARγ activation upregulated UCP2 expression in VSMCs; PPARγ inhibited PDGF-BB-induced ROS in VSMCs by upregulating UCP2 expression; PPARγ ameliorated injury-induced oxidative stress and intimal hyperplasia (IH) in UCP2-dependent manner. In conclusion, our study provides evidence that activation of PPARγ can attenuate ROS and VSMC proliferation and migration by upregulating UCP2 expression, and thus inhibit IH following carotid injury. These findings suggest PPARγ may represent a prospective target for the prevention and treatment of IH-associated vascular diseases.

## Introduction

Increasing evidence showed that abnormal proliferation and migration of vascular smooth muscle cells (VSMCs) are common event in the pathophysiology of many vascular diseases, including atherosclerosis and restenosis after angioplasty [1, 2]. In healthy blood vessel, VSMCs reside within the media and remain quiescent. In response to atherogenic factors, VSMCs proliferate and migrate from the media to the intima, which ultimately leads to intimal hyperplasia (IH) and vascular stenosis [3]. Animal studies showed that inhibiting proliferation and migration of VSMCs could attenuate IH significantly [4, 5]. Among the underlying mechanisms, oxidative stress is one of the principal contributors to the proliferation and migration of VSMCs [6].

Oxidative stress is defined as an imbalance between endogenous antioxidants and oxidants in favor of the later. Oxidative stress occurs as a result of persistent production of reactive oxygen species (ROS), which was reported to accelerate the proliferation and migration of VSMCs [7, 8]. Our previous study demonstrated that inhibition of ROS generation could attenuate the proinflammatory and proliferative phenotype of VSMCs [6]. Thereby, antioxidant treatment may be helpful in inhibiting the proliferation and migration of VSMCs. Under this context, recent research progress of the relationship between peroxisome proliferator-activated receptor γ (PPARγ) and oxidative stress provides new strategies for this phenomenon. Study of Chan et al. showed the protective effect of PPARγ activation against oxidative stress in the rostral ventrolateral medulla [9]. In addition, our and other previous studies also reported that PPARγ activation can modulate VSMC phenotype and reduce IH [10, 11]. However, it remains unclear whether activation of PPARγ can attenuate oxidative stress and further inhibit VSMC proliferation and migration or not.

In addition to xanthine oxidase, cyclo- and lipo-oxygenases, uncoupled endothelial nitric oxide synthase, peroxidases and NADPH oxidases [12], mitochondrial enzymes are the major source of the cellular ROS-generating enzymes [13, 14]. Mitochondrial uncoupling proteins (UCPs) have emerged as primary antioxidants to reduce oxidative stress and prevent oxidative damage by the maintenance of ROS homeostasis [15]. Among the members of UCPs, UCP2 is considered to be important to protect against oxidative stress [9, 16, 17]. Moreover, activation of PPARγ against oxidative damage in aging cerebral arteries is involved with upregulating UCP2 [18].

In this study, we tested the ROS production in PDGF-BB-induced VSMC proliferation and migration, and the inhibitory effect of PPARγ on this process. Using UCP2^-/-^ VSMC, we clarified the involvement of UCP2 in decreased oxidative stress and VSMC viability by PPARγ. Finally, our study provided evidence that activation of PPARγ could attenuate ROS production and VSMCs proliferation and migration by upregulating UCP2 expression, and thus attenuated IH following carotid injury.

## Materials and Methods

### Reagents

Dulbecco’s modified Eagle’s medium (DMEM), phosphate buffer solution (PBS) and fetal bovine serum (FBS) were purchased from Gibco BRL (Carlsbad, CA, USA). Recombinant murine platelet-derived growth factor-BB (PDGF-BB), 3-(4,5-dimethylthiazol-2-yl)-2,5-diphenyltetrazolium bromide (MTT), Dihydroethidium (DHE), GW9662 and N-acetylcysteine (NAC) were obtained from Sigma-Aldrich (St Louis, MO, USA). Rosiglitazone (RSG) was from Cayman (Ann Arbor, MI, USA). Antibodies targeting UCP2, PPARγ and β-actin were from Santa Cruz Biotechnology (Santa Cruz, CA, USA). Antibody against proliferating cell nuclear antigen (PCNA) was from Cell Signaling Technology (Beverly, MA, USA). Antibody targeting matrix metalloproteinase 9 (MMP9) was from Abcam (Burlingame, CA, USA).

### Animals

Male UCP2-deficient mice (C57BL/6J background) and wild-type mice (WT, C57BL/6J), 8–10 weeks of age, were purchased from the Jackson Laboratory (Bar Harbor, ME, USA). Carotid IH model was established by wire-induced carotid injury as described previously [19]. To determine the effects of the activated PPARγ on IH *in vivo*, PPARγ agonist RSG (10 mg/kg) was administrated intragastrically to WT and UCP2^-/-^ mice for 14 days starting from the day of wire injury. WT mice were randomized into three groups: sham operation group, injury group and injury group receiving RSG intragastrically. The fourth group was injury group from UCP2^-/-^ mice receiving RSG intragastrically. For surgery, mice were anaesthetized with an intraperitoneal injection of sodium pentobarbital (60 μg/g), and repeated injections (12 μg/g) were given as needed to maintain anesthesia. Carotid arteries in mice were harvested and examined 2 weeks after wire injury.

All the mice were housed in a specific pathogen free animal facility and maintained on a controlled light cycle schedule of 12:12 hour (h, light/dark) at 25°C with food and water *ad libitum*. Before experiments, the health inspection of animals was performed in experimental animal center according to the NIH guide for care and use of laboratory animals. After operation, the animals were kept warm and observed every half an hour, until they woke up and received water. Unexpected deaths and signs of illness, distress, or other deviations from normal animals would be found and investigated. Euthanize was used at the end of experimental process or to relieve pain or distress that cannot be alleviated by some treatments including anti-infection and adding nutrition dietary. The final performance was made by the professional in experimental animal center. The method of euthanasia was overdose administration of sodium pentobarbital by intraperitoneal injection. Animal care and procedures were conformed with the Guide for the Care and Use of Laboratory Animals. Protocol approval was from the Animal Research Committee of the Third Military Medical University.

### Histopathology

Animals were euthanized and carotid arteries were perfusion-fixed *in situ* with 4% formaldehyde solution and then harvested. Perfusion-fixed vessels were embedded in paraffin and sectioned with 6 μm thickness. Hematoxylin and eosin stain was used for pathological analysis. The area of each vascular layer was measured by tracing the external elastic lamina (EEL), internal elastic lamina (IEL) and vessel lumen. Intimal area was determined by subtracting the lumen area from the area within the IEL. Media area was considered as the area between the EEL and IEL. The IH was assessed by calculating the ratio of intima to media area. All the required reagents were obtained from Lab Vision (Fremont, CA, USA). Images were recorded by the Tissue Gnostics microscope (Zeiss, Oberkochen, Germany).

### Cell Culture

VSMCs were isolated from the thoracic aorta explants of WT or UCP2^-/-^ male mice(6–8 weeks) [20]. When the cells formed a confluent monolayer (10–14 days), then were passaged and cultured in DMEM supplemented with 10% FBS, penicillin (100 U/ml) and streptomycin (100 μg/ml) in humidified atmosphere of 95% air and 5% CO_2_ at 37°C. The cultured VSMCs were verified by targeting α-SMA using immunofluorescence. Second- to sixth-generation cells were selected for the experiments. To determine the effect of activated PPARγ on PDGF-BB induced proliferation of VSMCs, WT VSMCs were incubated with RSG (10 μmol/l) or together with GW9662 (antagonist of PPARγ, 5 μmol/l). UCP2^-/-^ derived VSMCs were used to determine the effect of UCP2.

### Western Blot

Western blot was used to analyze the protein levels of UCP2 and PPARγ. Protein samples were obtained from mouse aorta or cultured VSMCs. Protein samples were electrophoresed through a 12% sodium dodecyl sulfate polyacrylamide gel electrophoresis (SDS-PAGE) and transferred to a PVDF membrane. The membrane was incubated with anti-UCP2 (1:1000), anti-PPARγ (1:1000), anti-PCNA (1:500), anti-MMP9 (1:500) and anti-β-actin (1:2000) primary antibodies overnight at 4°C. After washing, the membrane was incubated with horseradish peroxidase-conjugated secondary antibodies for 2 h at room temperature. Then, proteins were detected by enhanced chemiluminescence (NEN, MA, USA) and quantified using a Gel Doc 2000 Imager (Bio-Rad, CA, USA). Western blot quantification was performed by densitometry and normalized to β-actin.

### Determination of ROS

ROS in carotid arteries and cultured VSMCs was measured using the method described previously [21, 22]. In brief, the fresh carotid arteries were isolated and embedded in tissue-freezing OCT compound, then were cut into 10-μm-thick sections. The artery sections and cultured VSMCs were placed on glass slides. DHE, dissolved in dimethyl sulfoxide (DMSO, 40 μmol/l), was added to the artery sections and cultured VSMCs and incubated at 37°C for 45min in the dark. After washing three times with PBS, the glass slides were placed under an inverted fluorescence microscope (Leica, German) and the fluorescence intensity was analyzed (Image-Pro Plus 6.0).

### Immunofluorescence

VSMCs on sterile glass cover slips were fixed with 4% paraformaldehyde. After fixation, the cells were permeabilized with Triton X-100 and blocked with 1% bovine serum albumin. Then, cells were incubated with anti-UCP2 antibody (1:100) overnight at 4°C. After washing in PBS, samples were incubated with FITC-conjugated secondary antibodies (1:100) for 1 h. Nuclear was stained with DAPI (4’6-diamidino-2-phenylindole, 10 mg/ml). The samples were observed under fluorescence microscope (Nikon, Japan).

### Cell Proliferation Assay

The MTT cell proliferation assay was used to observe VSMCs proliferation. The cells (3×10^4^ cells/ml) were plated on 96-well plates and incubated with PDGF-BB (20 μg/l) for 24 h with or without RSG. Then cell samples were incubated with a 20μl aliquot of 5 mg/ml MTT dye at 37°C for 4 h, then the cells were mixed thoroughly with 200 μl DMSO. Light absorbance (570 nm) was measured with microplate reader (Bio-Rad, CA, USA). Besides the MTT assay, western blot was also used to detect the expression of PCNA (common marker for cell proliferation)following the detailed procedures mentioned above.

### Cell Migration Assay

Boyden chambers were used to measure VSMC migration. Briefly, the cells (5×10^4^ cells/mL) were plated in the upper chamber in serum-free DMEM with or without RSG, while DMEM containing 0.4% FBS and PDGF-BB (20 μg/l) were placed in the chamber below. The samples on the underside of the filters were fixed with methanol and stained with hematoxylin after incubation for 24 h at 37°C. At last, migrating cells were counted from five high-power fields (×400) per well. Besides the Boyden chamber assay, we also used western blot to detect the expression of MMP9 (common marker for cell migration), and the detailed procedures were mentioned above.

### Statistical Analysis

Data were expressed as the means ± SEM of at least three independent experiments. Multiple-group statistical analyses were performed by one-way ANOVA followed by least significant difference post hoc testing. Statistics were calculated with the GraphPad Prism 5 software package (GraphPad Software, La Jolla, CA, USA). Differences were considered statistically significant at P<0.05.

## Result

### PDGF-BB-Stimulated-VSMC Showed Increased Proliferation and Migration as well as ROS

**Fig 1:**
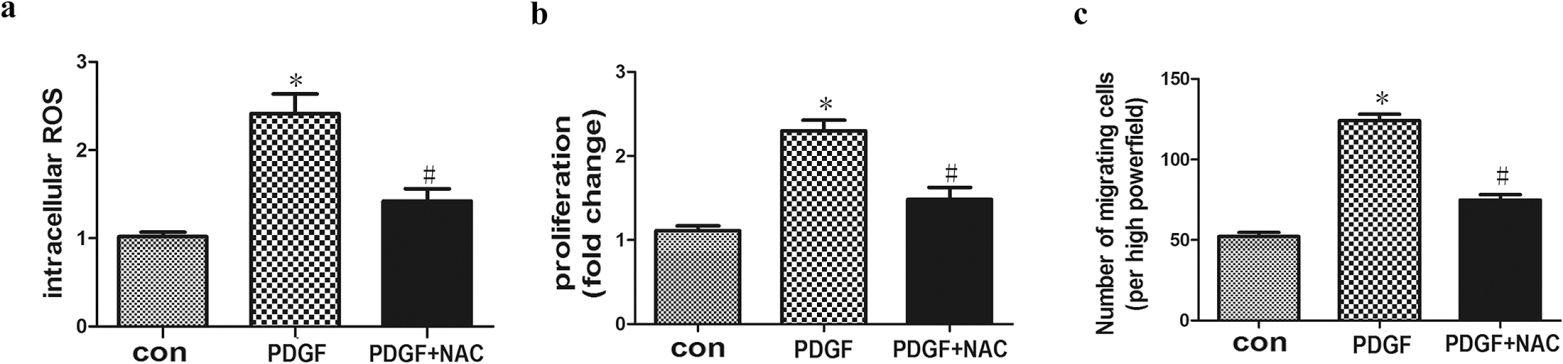
PDGF-BB-stimulated-VSMC showed increased proliferation and migration as well as ROS. VSMC from WT mice in basal conditions displayed low concentration of ROS production, PDGF-BB (20 μg/l) treatment significantly elevated ROS level (a). Proliferation and migration of VSMC showed significant increase in response to PDGF-BB compared with con group (b and c). NAC (10mmol/l) significantly reduced the intracellular ROS production and VSMC proliferation and migration induced by PDGF-BB. (*P<0.05 *vs*. con; *#P*<0.05 *vs*. PDGF). Con, wild-type VSMCs in basal conditions; PDGF, PDGF-BB, platelet derived growth factor-BB; NAC, N-acetylcysteine; ROS, reactive oxygen species.

PDGF-BB is traditionally used to induce the cultured VSMC proliferation and migration. In the present study, PDGF-BB (20 μg/l) was added in the cultured VSMCs from WT mice. Then intracellular ROS concentration was measured by fluorescence using DHE, and VSMC proliferation and migration were tested by MTT assay and Boyden chambers. The present study showed that PDGF-BB treatment significantly induced oxidative stress manifested by elevated ROS level in cultured VSMCs (Fig 1a). In addition, VSMCs from WT mice showed significant increase of proliferation and migration in response to PDGF-BB (Fig 1b and 1c). NAC (10mmol/l) significantly reduced the intracellular ROS production and VSMC proliferation and migration induced by PDGF-BB (Fig 1). Therefore, these results indicate that PDGF-BB-stimulated-VSMC increased proliferation and migration as well as ROS.

### PPARγ Inhibited PDGF-BB-Induced VSMC Proliferation, Migration and Oxidative Stress

**Fig 2:**
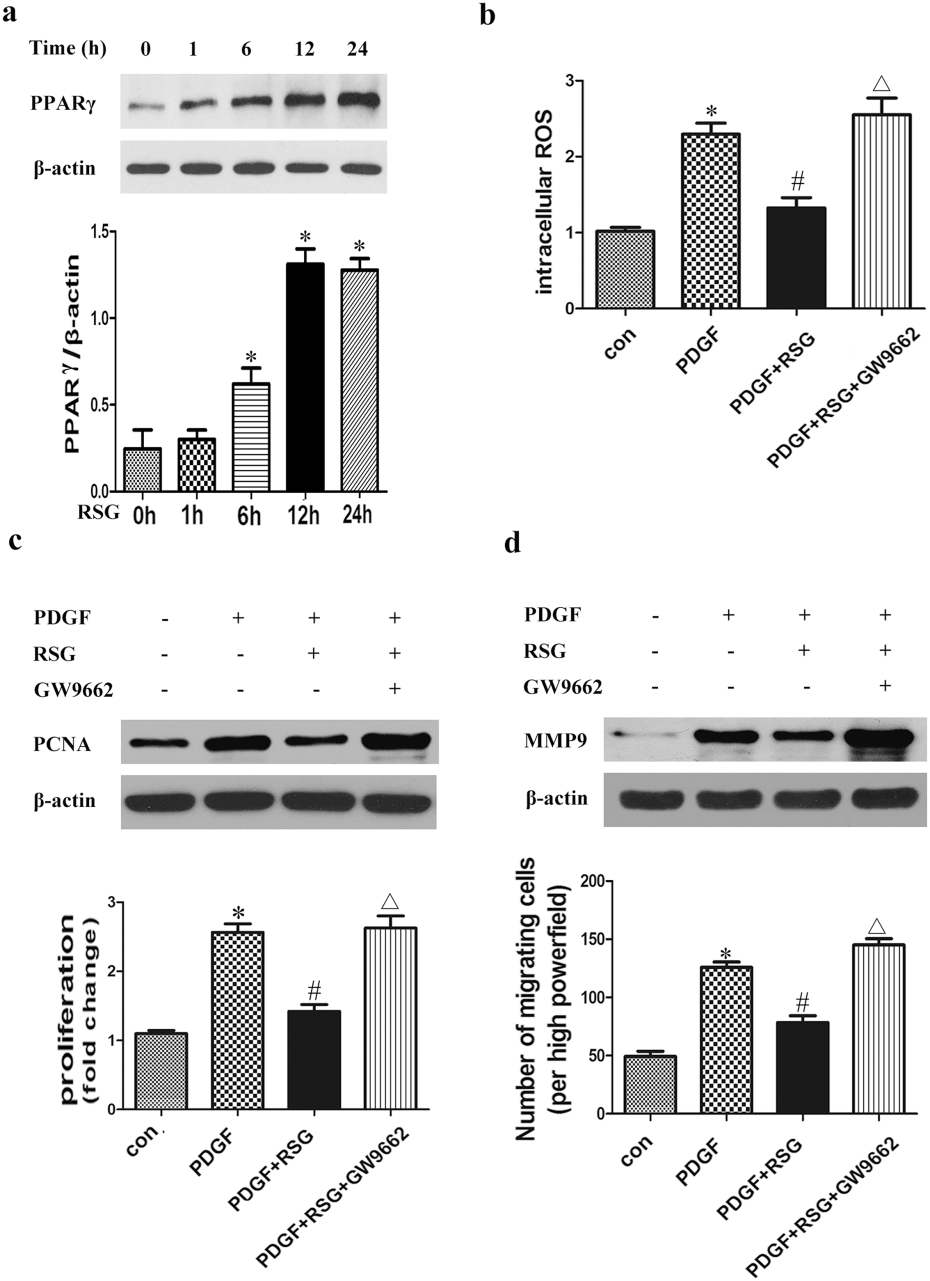
PPARγ inhibited PDGF-BB-induced VSMC proliferation, migration and oxidative stress. Cultured VSMCs were incubated with RSG (10μmol/L) for different times (0, 1, 6, 12 or 24 h). PPARγ expression increased in time-dependent manner, with an obvious effect at 6 h and the peak at 12 h that sustained after 24 h (a). (*P<0.05 *vs*. 0 h). RSG treatment for 12 h significantly counteracted ROS generation induced by PDGF-BB in VSMCs, while PPARγ inhibitor GW9662 diminished the effect of RSG (b). VSMCs treated with RSG showed reduced proliferation and PCNA expression, VSMC migration and MMP9 expression in response to PDGF-BB, which was reversed by GW9662 (c and d). (*P<0.05 *vs*. con; #*P*<0.05 *vs*. PDGF; Δ*P*<0.05 *vs*. PDGF+RSG). Con, wild-type VSMCs in basal conditions; PDGF, PDGF-BB, platelet derived growth factor-BB; ROS, reactive oxygen species; RSG, rosiglitazone; GW9662, PPARγ inhibitor; PCNA, proliferating cell nuclear antigen; MMP9, matrix metalloproteinase 9.

RSG, a PPARγ specific ligand, can activate and upregulate PPARγ in VSMCs. Western blot was used to measure the expression of PPARγ in VSMCs in response to RSG (10 μmol/l) stimulation for different time periods. It was found that RSG upregulated the PPARγ expression, with the peak reached at 12 h and sustained after 24 h (Fig 2a). Our study found that PPARγ activation by RSG for 12 h significantly counteracted ROS generation in VSMCs, and thus mitigated PDGF-BB-induced oxidative stress, while PPARγ inhibitor GW9662 (5 μmol/l) diminished the effect of RSG (Fig 2b). VSMCs treated with RSG showed reduced proliferation and PCNA (common marker for cell proliferation) expression in response to PDGF-BB, which was reversed by GW9662 (Fig 2c). Consistently, activation of PPARγ reduced VSMC migration and MMP9 (common marker for cell migration) expression in response to PDGF-BB, which was reversed by GW9662 (Fig 2d). These data suggest that activation of PPARγ inhibited PDGF-BB-induced VSMC proliferation, migration and ROS production.

### PPARγ Activation Upregulated UCP2 Expression in VSMCs

**Fig 3:**
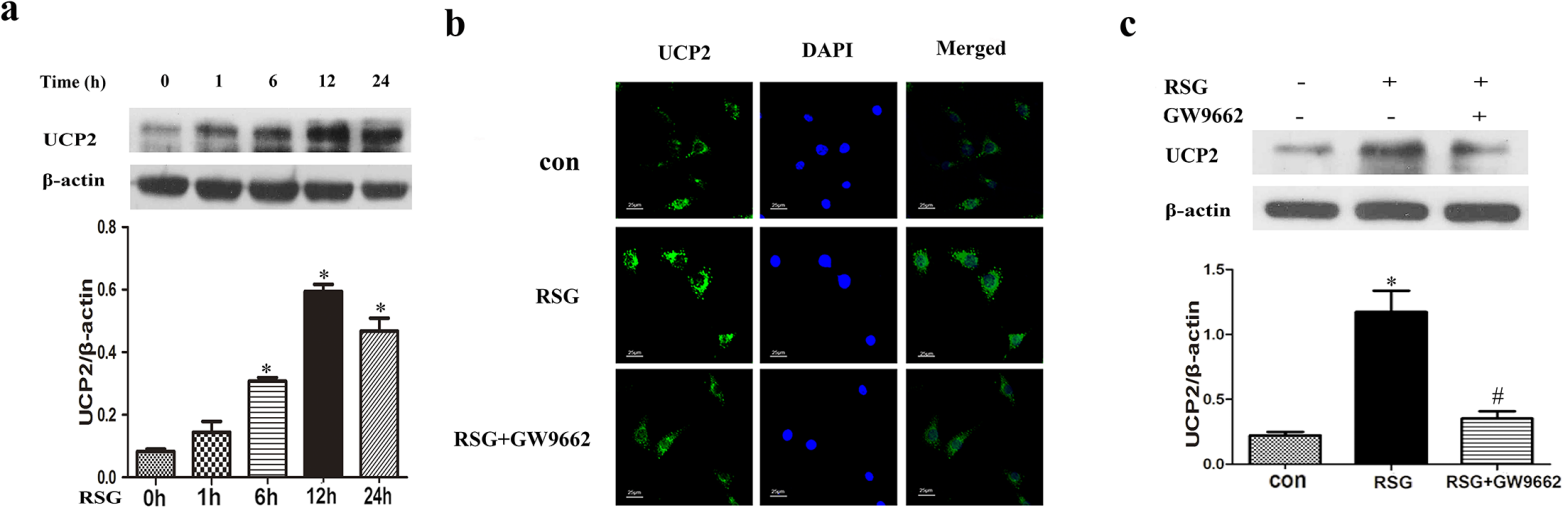
PPARγ activation upregulated UCP2 expression in VSMCs. Cultured VSMCs were incubated with RSG (10 μmol/l) for different times (0, 1, 6, 12 or 24 h). The level of UCP2 expression increased in time-dependent manner with an obvious effect at 6 h and the peak at 12 h that sustained after 24 h (a). (*P<0.05 *vs*. 0 h). RSG (10 μmol/l) treatment significantly upregulated the UCP2 expression which was reversed by PPARγ inhibitor GW9662 (5 μmol/l) detected respectively by immunofluorescence (b, green-UCP2, blue-DAPI) and western blot (c). (*P<0.05 *vs*. con; #*P*<0.05 *vs*. RSG). Con, wild-type VSMCs in basal conditions; PDGF, PDGF-BB, platelet derived growth factor-BB; ROS, reactive oxygen species; UCP2, uncoupling protein 2; RSG, rosiglitazone.

Mitochondria are the main source of ROS. UCP2, a mitochondrial protein, plays an important role in feedback mechanisms controlling the production of mitochondrial ROS. Previous study showed that activated PPARγ ameliorated oxidative stress in the rostral ventrolateral medulla by upregulating UCP2 [9]. In the present study, PPARγ activation by RSG (10 μmol/l) increased UCP2 expression in time-dependent manner, with the peak at 12 h (Fig 3a). PPARγ inhibitor GW9662 (5μmol/l) diminished the effect of RSG on green fluorescent protein-tagged UCP2 expression and UCP2 protein level were observed respectively by immunofluorescence and western blot (Fig 3b and 3c).

### PPARγ Inhibited PDGF-BB-Induced ROS in VSMCs by Upregulating UCP2 Expression

**Fig 4:**
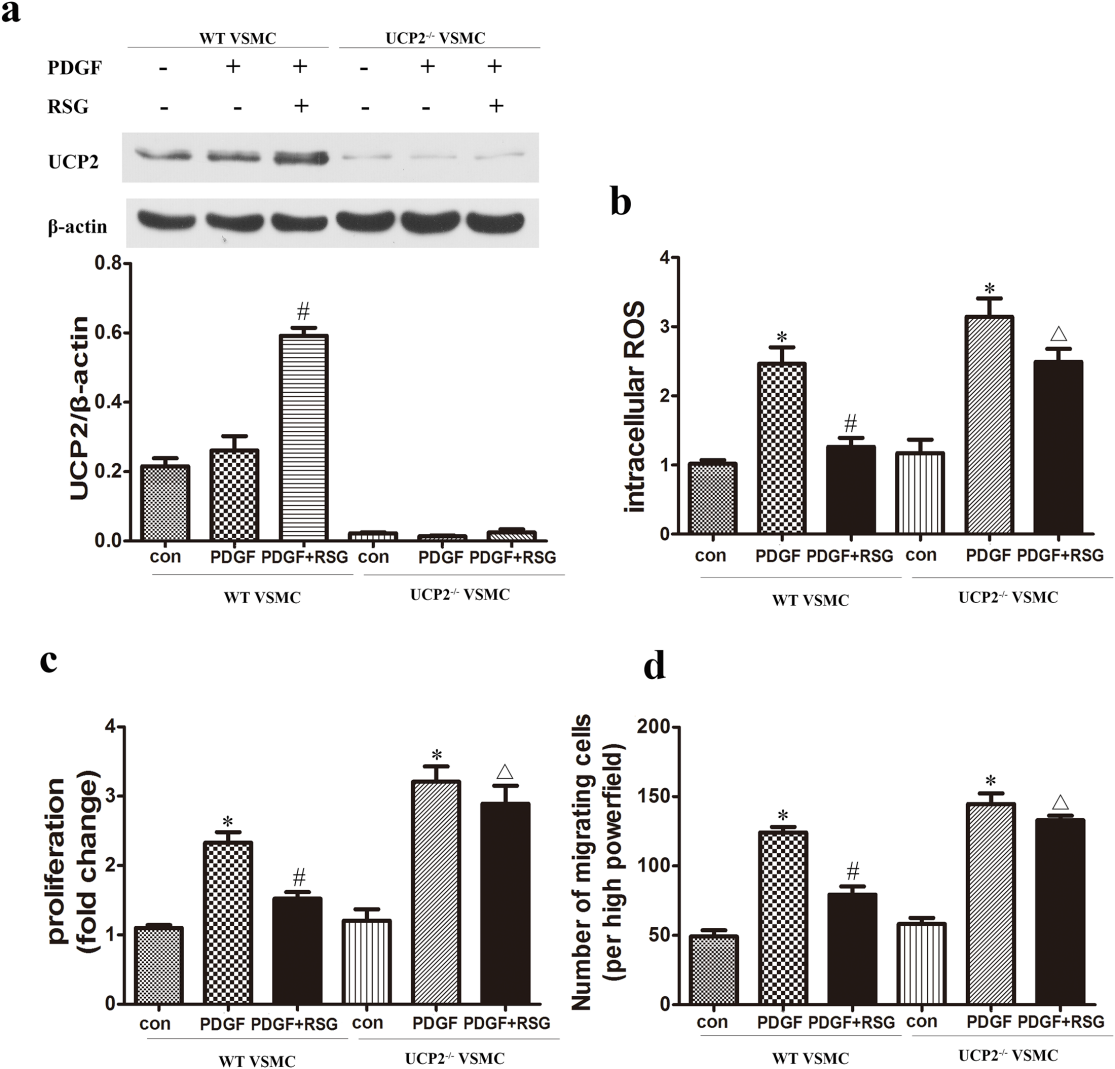
PPARγ inhibited PDGF-BB-induced ROS in VSMCs by upregulating UCP2 expression. UCP2 expression in cultured WT- and UCP2^-/-^ − VSMCs in response to PDGF-BB (20 μg/l) and RSG (10 μmol/l) was detected by western blot (a). Compared to WT-VSMCs, UCP2^-/-^ -VSMCs displayed much higher ROS level (b), enhanced proliferation (c) and migration (d) in response to PDGF-BB. The RSG treatment failed to impeded the PDGF-induced ROS production and VSMC proliferation and migration in UCP2^-/-^ -VSMCs (b-d). (*P<0.05 *vs*. con; #*P*<0.05 *vs*. PDGF; Δ*P*<0.05 *vs*. WT-VSMCs treated with PDGF and RSG). Con, wild-type VSMCs in basal conditions; ROS, reactive oxygen species; UCP2, uncoupling protein 2; RSG, rosiglitazone; UCP2^-/-^ VSMC, VSMCs from UCP2^-/-^ mice; WT-VSMC, VSMCs from wild-type mice.

UCP2^-/-^ mice derived VSMCs were used to identify the role of UCP2 in PDGF-BB-induced intracellular ROS production and cell proliferation and migration. We measured UCP2 expression and ROS level in response to PDGF-BB in VSMCs treated with or without RSG. It was shown that RSG activating PPARγ significantly increased UCP2 expression (Fig 4a) and diminished the PDGF-BB-induced ROS elevation (Fig 4b) in WT-VSMCs. However, UCP2^-/—^VSMCs showed low level of UCP2 (almost undetected), displayed much higher ROS level in response to PDGF-BB, and RSG treatment failed to upregulate UCP2 expression and impeded the PDGF-induced ROS elevation in UCP2^-/—^VSMCs (Fig 4b), indicating that UCP2 is required in PPARγ-mediated ROS reduction. Consistently, UCP2^-/—^VSMCs displayed much higher proliferation and migration in response to PDGF-BB, which were not impeded by RSG treatment (Fig 4c and 4d), indicating the essential role of UCP2 in the inhibitory effect of PPARγ on VSMC proliferation and migration.

### PPARγ Ameliorated Injury-Induced Oxidative Stress and Intimal Hyperplasia in UCP2-Dependent Manner

**Fig 5:**
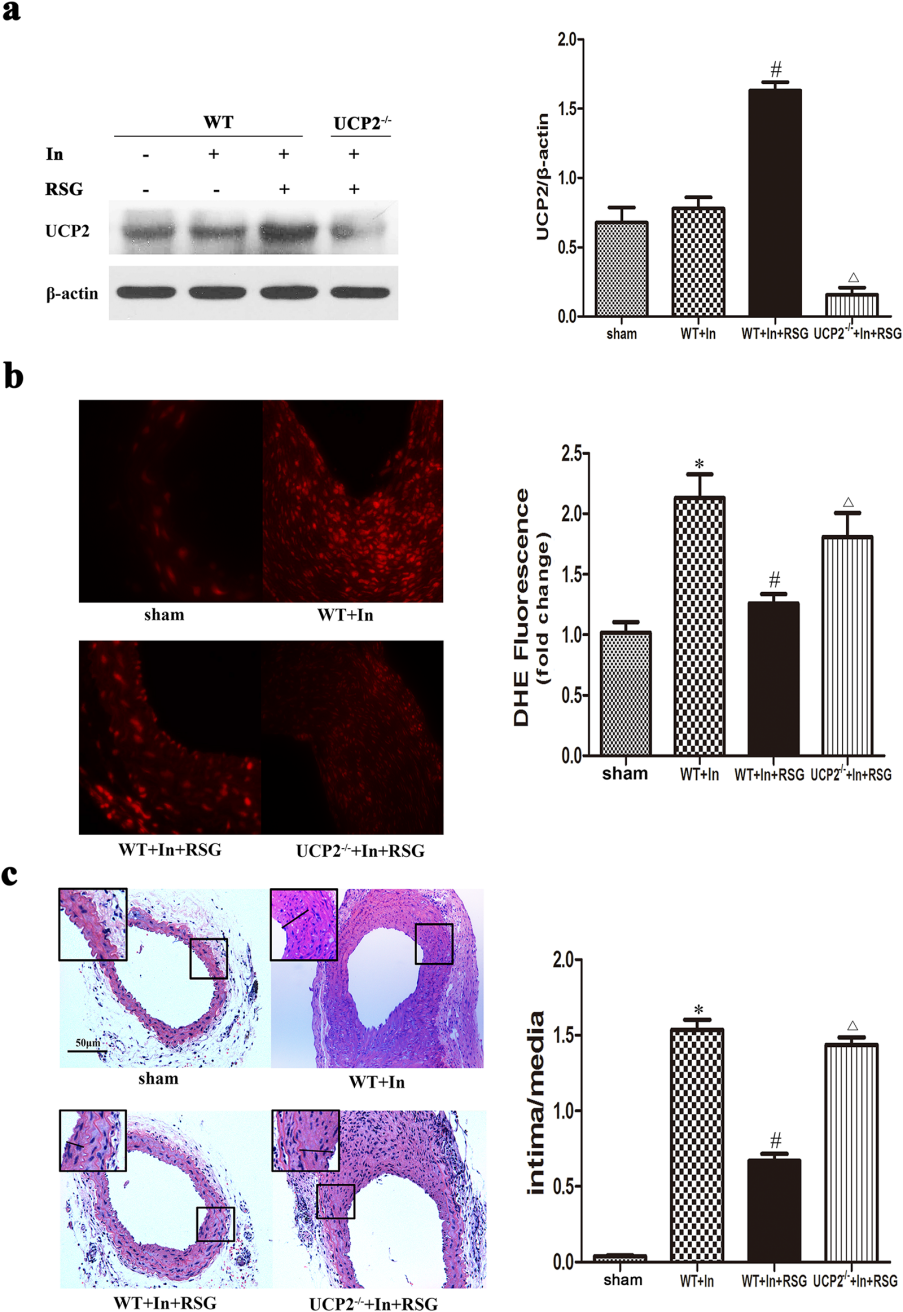
PPARγ ameliorated injury-induced oxidative stress and intimal hyperplasia in UCP2-dependent manner. RSG significantly increased UCP2 expression in injured arteries (In) from WT mice but not in UCP2^-/-^ mice (a). Carotid arteries ROS was detected by DHE staining (b). The increased ROS by wire injury was also reduced by RSG in WT mice but not in UCP2^-/-^ mice. Hematoxylin and eosin staining on cross-sections from representative injured carotid arteries are presented (c). Carotid wire injury induced IH with increased intima/media ratio in WT mice. RSG inhibited the wire-injury-induced IH in WT mice but not in UCP2^-/-^ mice. (*P<0.05 vs. sham; #*P*<0.05 vs. WT+In; Δ*P*<0.05 vs. WT+In+RSG). Sham, sham operation; WT, wild-type mice; In, carotid wire injury; ROS, reactive oxygen species; RSG, rosiglitazone; UCP2^-/-^, UCP2-dificient mice.

For *in vivo* study, wire-injury in carotid arteries was used to induce IH. Fourteen days after surgery, RSG significantly increased UCP2 expression in injured arteries from WT mice (Fig 5a). We assessed the IH by calculating the ratio of intima to media area. The increased ROS by wire injury was also reduced by RSG in WT mice but not in UCP2^-/-^mice (Fig 5b). As shown in Fig 5c, the injured carotid artery showed significant IH, manifested by increased intima/media ratio compared with the arteries from sham-operated mice (1.5360±0.0542 *vs* 0.0385±0.0048, P<0.05). Oral RSG (10 mg/kg) significantly attenuated the wire injury-induced IH (0.6695±0.0374 *vs* 1.5360±0.0542, P<0.05). In contrast, RSG failed to effectively impede the wire injury-induced IH in UCP2^-/-^ mice (1.4360±0.0403 *vs* 1.5360±0.0542, P>0.05). Together, these results show that PPARγ ameliorates injury-induced oxidative stress and IH required by UCP2.

## Discussion

In addition to the physiological role in controlling endothelial function and vascular tone, ROS modulates the inflammatory response, cell proliferation, migration and apoptosis, contributing to vascular remodeling and endothelial dysfunction [23]. Excessive vascular production of ROS or oxidative stress involves in various chronic diseases. VSMC proliferation and migration exert an important role in IH which contributes to restenosis or occlusion after vascular procedures. Although the positive role of activation of PPARγ in regulating VSMC proliferation and migration and IH has been well-accepted, the underlying mechanisms remain unclear. The present study aimed to confirm the role of PPARγ against oxidative stress, to further raise the prospect of PPARγ in improving VSMC functions and inhibiting IH.

Our data demonstrated that PDGF-BB-stimulated-VSMC showed increased proliferation and migration as well as ROS. Activation of PPARγ increased UCP2 expression and inhibited ROS production, proliferation and migration of VSMCs; while UCP2 deficiency diminished the effect of PPARγ on reducing ROS production and proliferation and migration of VSMCs. Activation of PPARγ by oral RSG upregulated UCP2 expression and attenuated IH and oxidative stress after wire-induced carotid injury.

ROS are species including superoxide anion, hydroxyl radicals, and hydrogen peroxide. They are the destructive aspect of oxidative stress. Increasing evidence suggest that ROS participate in various vascular cell signaling by modulating redox-sensitive transcription and transduction pathways [24]. Previous study showed that high level of ROS was observed in intimal VSMCs of hyperplastic and atherosclerotic lesions [25]. In addition, numerous studies have shown that ROS accumulation played a critical role in the proliferation and migration of VSMCs or even IH [26, 27]. Our data demonstrated that ROS production was markedly elevated as well as the enhanced proliferation and migration in PDGF-BB-treated VSMCs, which was reversed when treated by ROS inhibitor NAC. Thereby, attenuating ROS production may hold promising therapeutic target for preventing the proliferation and migration of VSMCs in process of IH.

PPARγ, a member of PPAR superfamily, is mainly expressed in adipocytes and activates both lipogenic and lipid oxidation [28]. Reportedly, PPARγ activation attenuated ROS production in aging rat cerebral arteries and human umbilical vein endothelial cells [29]. Our previous study showed that PPARγ attenuated carotid arteries IH by inhibiting TLR4-mediated inflammation in VSMCs [11]. Yang et al. showed that activation of PPARγ by RSG inhibited VSMC phenotype via a protein kinase G-dependent pathway and reduced IH after vascular injury [10]. Wan et al. implied that activation of PPARγ prevented the VSMCs proliferation via modulation of caspase and cyclin signaling pathways [30]. However, whether activation of PPARγ can influence oxidative stress and thereby inhibit VSMC proliferation and migration is currently unknown. Our study demonstrated that activation of PPARγ by RSG significantly reduced the production of ROS induced by PDGF-BB. Meanwhile, the proliferation and migration of VSMCs were also inhibited. As expected, PPARγ inhibitor GW9662 diminished the effect of RSG. In addition, our *in vivo* study also showed that oral intake of RSG significantly reduced the ROS level of injured carotid arteries, and attenuated neointimal formation induced by wire injury. Taken together, the data suggest that activation of PPARγ by RSG inhibited proliferation and migration of VSMCs and IH by attenuating oxidative stress. However, as oxidative stress is the result of unbalanced production of antioxidants and oxidants, we wonder that how activation of PPARγ exerts its antioxidant effect.

UCPs are proteins in the mitochondrial inner membrane. They belong to a superfamily of mitochondrial anion transporters that uncouple ATP synthesis from oxidative phosphorylation by controlling the leakage of protons across the inner mitochondrial membrane [31]. UCPs have become an important natural antioxidants in the maintenance of ROS homeostasis, apart from scavenging enzymes (e.g. superoxide dismutase and catalase) and low molecular weight antioxidants (e.g. ascorbic acid and glutathione) [32, 33]. By now, five subunits of UCPs have been found in mammals: UCP1-UCP5, among which, UCP2 distributes widely in several tissues and has been widely studied [34, 35]. Numerous studies have reported that increased UCP2 expression could attenuate oxidant tissue damage [36, 37]. Chan et al. showed that activation of PPARγ by RSG significantly increased the expression of UCP2 and reduced mitochondrial hydrogen peroxide level in rostral ventrolateral medulla [9]. Chuang et al. showed that activation of PPARγ by RSG enhanced UCP2 expression and further protected against oxidative stress and neuronal cell death associated with cerebral ischemia [38]. Wang et al. showed that activation of PPARγ by pioglitazone attenuated oxidative stress in aging rat cerebral arteries through upregulating UCP2 [29]. However, it is still unknown whether PPARγ exerts antioxidant effect through upregulating UCP2 in PDGF-BB or wire injury induced VSMC proliferation and migration. Our data demonstrated that activation of PPARγ by RSG significantly increased the expression of UCP2, which was abolished by the addition of PPARγ antagonist GW9662. Similar results were also found in *in vivo* study that oral intake of RSG significantly increased the expression of UCP2 of injured carotid arteries.

To further explore the role of UCP2 in the antioxidant effect of PPARγ, we used UCP2^-/-^ mice and UCP2^-/-^ derived VSMCs. In cultured VSMCs, PDGF-induced increased ROS, proliferation and migration were significantly reversed by PPARγ activation. However, in UCP2^-/-^ derived VSMCs, the inhibitory effects of PPARγ activation on ROS production and proliferation and migration was diminished. Similarly in *in vivo* study, oral intake of RSG significantly reduced the ROS level of injured carotid arteries and attenuated IH induced by wire injury. But all these were not shown in UCP2^-/-^ mice. Taken together, our data suggest that activation of PPARγ attenuates oxidative stress and inhibits VSMCs proliferation and migration through upregulating UCP2.

The precise mechanisms involved in the regulation of PPARγ on UCP2 expression are still unclear, given that no PPAR response element (PPRE) within or near the UCP2 gene has been annotated [17]. Previous study showed that PPARγ does not bind to the promotor region of UCP2 gene, suggesting that PPARγ regulates the UCP2 through an indirect mechanism and likely requires other transcription factors [39]. Bugge’s study proposed that the PPARγ/RXR (retinoid X receptor) binding site in intron 1 of UCP3 gene facilitates PPARγ transactivation of the promotor of UCP2 gene [40]. Due to the limited reports regarding mechanism of PPARγ regulating UCP2, we did not make exploration on this issue, and this is a limitation in the design of this study. Moreover, we detected the production of total ROS instead of mitochondrial ROS in VSMCs and tissues. This is another defect in our experiment, which is needed to be studied in our future investigation.

In conclusion, our study provides evidence that activation of PPARγ can attenuate ROS production and VSMC proliferation and migration by upregulating UCP2 expression, and thus attenuate IH following carotid injury. These findings suggest PPARγ may represent a prospective target for the prevention and treatment of IH-associated vascular diseases.
